# ^1^H-NMR-based metabolomics reveals metabolic alterations in early development of a mouse model of Angelman syndrome

**DOI:** 10.1186/s13229-024-00608-2

**Published:** 2024-07-24

**Authors:** Pooja Kri Gupta, Sharon Barak, Yonatan Feuermann, Gil Goobes, Hanoch Kaphzan

**Affiliations:** 1 Sagol Department of Neurobiology, Faculty of Natural Sciences, University of Haifa, Haifa, 3103301 Israel; 2 Department of Chemistry and The Institute for Nanotechnology and Advanced Materials, Bar-Ilan University, Ramat Gan, 5290002 Israel

**Keywords:** Angelman syndrome, Metabolite, Mitochondria, Reactive oxygen species, Developmental disorders, Lactate, Acetate, Succinate, Glycolysis, Pyruvate metabolism

## Abstract

**Background:**

Angelman syndrome (AS) is a rare neurodevelopmental genetic disorder caused by the loss of function of the ubiquitin ligase E3A (*UBE3A*) gene, affecting approximately 1:15,000 live births. We have recently shown that mitochondrial function in AS is altered during mid to late embryonic brain development leading to increased oxidative stress and enhanced apoptosis of neural precursor cells. However, the overall alterations of metabolic processes are still unknown. Hence, as a follow-up, we aim to investigate the metabolic profiles of wild-type (WT) and AS littermates and to identify which metabolic processes are aberrant in the brain of AS model mice during embryonic development.

**Methods:**

We collected brain tissue samples from mice embryos at E16.5 and performed metabolomic analyses using proton nuclear magnetic resonance (^1^H-NMR) spectroscopy. Multivariate and Univariate analyses were performed to determine the significantly altered metabolites in AS mice. Pathways associated with the altered metabolites were identified using metabolite set enrichment analysis.

**Results:**

Our analysis showed that overall, the metabolomic fingerprint of AS embryonic brains differed from those of their WT littermates. Moreover, we revealed a significant elevation of distinct metabolites, such as acetate, lactate, and succinate in the AS samples compared to the WT samples. The elevated metabolites were significantly associated with the pyruvate metabolism and glycolytic pathways.

**Limitations:**

Only 14 metabolites were successfully identified and investigated in the present study. The effect of unidentified metabolites and their unresolved peaks was not determined. Additionally, we conducted the metabolomic study on whole brain tissue samples. Employing high-resolution NMR studies on different brain regions could further expand our knowledge regarding metabolic alterations in the AS brain. Furthermore, increasing the sample size could reveal the involvement of more significantly altered metabolites in the pathophysiology of the AS brain.

**Conclusions:**

*Ube3a* loss of function alters bioenergy-related metabolism in the AS brain during embryonic development. Furthermore, these neurochemical changes could be linked to the mitochondrial reactive oxygen species and oxidative stress that occurs during the AS embryonic development.

**Supplementary Information:**

The online version contains supplementary material available at 10.1186/s13229-024-00608-2.

## Background

Angelman syndrome (AS) is a rare genetic neuropsychiatric disorder that manifests during the early child’s development and is characterized by severe developmental delay, happy demeanor, hyperactivity, motor dysfunction, absence of speech, and seizures [1, 2]. The prevalence of AS is ~ 1:15,000 live births [3]. AS is caused by the loss of function of the ubiquitin ligase E3A (UBE3A) protein, which stems in most cases from a deletion of the maternal chromosome 15q11.2-q13 region [4–6]. UBE3A, also known as E6-associated protein (E6AP), polyubiquitinates substrate proteins to target them for their degradation by the proteasome system [7, 8]. Under normal circumstances, the expression of the paternal *Ube3a* allele is silenced in neurons before postnatal day 7 in mice, and thereafter, consistent UBE3A levels are maintained through maternal allele expression. Therefore, with the loss of function of the maternal allele, UBE3A expression is almost absent in adult AS [9, 10]. However, in the late embryonic stages, when the paternal allele is expressed, AS mice are expected to have a 50% reduction in UBE3A expression [10, 11].

To study the pathophysiology of the syndrome, several mouse models for AS were generated over the years. These mouse models recapitulate the human AS disorder, as they exhibit multiple levels of brain dysfunction that resemble the human disorder including cognitive dysfunction, susceptibility to epilepsy, impaired motor function, and other behavioral abnormalities [12–15]. Almost all studies that investigated AS pathophysiology were performed in adult mice, and almost none investigated the embryonic development of the AS brain. In systematic studies to find out the developmental role of UBE3A, researchers have found that core autistic-related phenotypes were only rescued when *Ube3a* reinstatement was done at an early embryonic stage, while a later reinstatement was not successful in a complete alleviation of the AS-related phenotypes [10, 16, 17]. These results suggest that *Ube3a* is critical for normal embryonic brain development.

A few studies by us and others have pointed towards the contribution of mitochondrial dysfunction to the pathophysiology of AS [11, 18–22]. A study that investigated mitochondrial functioning in hippocampal neurons of adult Angelman syndrome model mice found small dense mitochondria with altered cristae and oxidative phosphorylation defects, with decreased complex III activity in the whole brain tissue [18]. Following up on this study, we have found that mitochondrial oxidative stress is associated with AS-related hippocampal deficits, and that reduction of mitochondrial oxidative stress using mitoQ resulted in improving hippocampal CA1 long-term potentiation and hippocampal-dependent memory [19]. Similarly, another study showed impaired mitochondrial respiration with reduced complex-III activity, which was restored by supplementing Idebenone, a CoQ10 analog [22]. This therapy also rescued the reduced marble-burying and rotarod deficits [22]. In a later study, we explored the impact of *Ube3a* deletion on mitochondrial functioning, using mouse embryonic fibroblasts (MEFs). This investigation identified alterations in mitochondrial-related genes and pathways at both transcriptomic and proteomic levels [20, 21]. Another study by us demonstrated dysregulation of mitochondrial function and oxidative stress at an embryonic stage in the Angelman syndrome mouse model [11]. Specifically, multifaceted mitochondrial aberrations were observed in AS brain-derived embryonic neural precursor cells (NPCs), including enhanced mitochondrial membrane potential, excessive levels of mitochondrial reactive oxygen species (ROS), and increased levels of apoptosis [11]. Especially, the enhanced level of mitochondrial superoxide in AS was accompanied by a slight reduction in total glutathione levels alongside a strong reduction in reduced glutathione levels. We further showed that apoptosis levels were rescued by supplementation of the antioxidant glutathione-reduced ethyl ester (GSH-EE). Moreover, the administration of GSH-EE not only lowered the enhanced apoptosis observed by TUNEL staining and caspase 3/7 activity but also lowered the BAX/BCL2 ratio, which is the relation between the two essential apoptotic regulators that act at the mitochondria, the pro-apoptotic BAX protein and the anti-apoptotic BCL-2 protein that was found elevated in AS NPCs [11]. Interestingly, the enhanced apoptosis that was found in E16.5 NPCs of AS mice was shown to result from the intrinsic apoptosis pathway, while the extrinsic pathway was found to be not affected given that caspase-8 activity was unaltered [11].

Mitochondria are well known for their role in cellular adenosine triphosphate (ATP) production, intracellular calcium signaling, and apoptosis, and are a major production site of cellular metabolites and ROS [23]. Thus, mitochondrial metabolism serves as a key regulator of embryonic development, controlling proliferation, neurogenesis, differentiation, and apoptosis [24]. As aforementioned, studies by us and others have shown a link between the *Ube3a* deletion and mitochondrial dysfunction [11, 18–22, 25].

Mitochondria being a metabolic hub regulate not only energy production processes but also other molecular pathways, thus affecting embryonic brain development [26, 27]. For example, glycolysis and the TCA cycle are major pathways providing metabolic precursors for biosynthesis and energy production. Mature neurons rely on oxidative phosphorylation for ATP during neonatal development, while neural precursor cells rely on anaerobic glycolysis [28–30]. This metabolic switch from anaerobic glycolysis to oxidative phosphorylation has been regarded as a hallmark of neuronal differentiation. Accuracy in mitochondrial functioning is required for healthy brain development and functioning. Mitochondrial metabolism alterations were found to be involved in multiple neurological disorders such as Alzheimer’s disease and autism [31, 32].

Given our previous studies that demonstrated mitochondrial dysfunction, increased oxidative stress, and enhanced apoptosis in neural precursor cells of AS mice embryos at E16.5, we hypothesized that the aberrant mitochondrial functioning during embryonic stages disrupts the metabolic profile of the brain cells, and we aimed to follow-up in delineating few of these metabolomic alterations. Additionally, because these metabolites play pivotal roles in essential cellular processes such as energy metabolism, antioxidant function, and mitochondrial respiration, we aimed to determine whether the expression levels of such metabolites are altered in the AS brain during early developmental stages. For that, we carried out metabolomic profiling using ^1^H-NMR spectroscopy and found marked differences in the levels of lactate, acetate, and succinate metabolites. Altered metabolite levels have also been observed in several other neurological disorders such as schizophrenia, down syndrome, and Parkinson’s disease [33–35]. Metabolic alterations of this nature have the potential to change the trajectory of brain cells and be the cause of the observed disruption of brain development in AS.

## Methods

### Animals

Twelve E16.5 embryos, 6 AS model mice (*Ube3a*^m−/p+^), and 6 WT (*Ube3a*^m+/p+^) embryonic littermates on the background strain of C57BL/6 AS were generated by breeding female mice with paternal deletion of *Ube3a* (*Ube3a*^m+/p−^) with WT male mice (*Ube3a*^m+/p+^), as previously described [36–38, 14, 39]. The WT littermates served as controls. The AS model line used is JAX stock #016590, and the animals were genotyped using primers as previously described [12]. The mice were group-housed on a 12 h light/dark cycle with *ad libitum* access to food and water. Housing, breeding, and all experimental procedures were conducted according to the guidelines of the University of Haifa Institutional Ethics Committee.

### Sample preparation for NMR analysis

On gestational day E16.5, embryos were surgically extracted on ice and the embryonic brains from the pregnant female mice were immediately harvested and snap-frozen in liquid nitrogen and stored at -80℃ until further processing. The extraction of metabolites was done as previously described by Zheng et al. [40]. In brief, the frozen tissue was weighed into an Eppendorf tube, and 4 ml/g of ice-cold methanol and 0.85 ml/g of distilled water were added to the tube for protein precipitation [41]. The mixture was homogenized at 4℃ and then vortexed. After homogenization, 2 ml/g of ice-cold chloroform and 2 ml/g of distilled water were added to the tube and vortexed. The mixture was kept on ice for 15 min and then centrifuged at 10,000 g for 15 min at 4℃ to achieve phase separation. Following centrifugation, the supernatant was transferred to a fresh tube and lyophilized for approximately 24 h. The lyophilized extract was stored at -80℃ until NMR analysis. The dried samples were re-constituted in 0.6 ml of 99.5% D2O containing 0.05% of the internal standard trimethylsilyl-2,2,3,3, -tetradeuteroproprionate (TSP) in a 5 mm NMR tube for metabolomic analysis.

### NMR measurement

All ^1^H NMR spectra were acquired using Bruker AVANCE III 500.04 MHz NMR spectrometer (Bruker BioSpin, Rheinstetten, Germany) at 300 K using a ^1^H one-pulse with water presaturation sequence, zgpr. Measurements were done using a QNP probe. The resolution of lines without processing was 1.72 Hz or 0.00348 ppm at the magnetic field (11.74 Tesla). The acquisition parameters included scans: 200; receiver gain: 1030.0; pulse width: 8.00 µs; acquisition time: 4.68 s; relaxation delay: 4.00 s; spectral width: 7002.801 Hz. Using these acquisition parameters, the measurement was quantitative.

### NMR data preprocessing, metabolite identification, and quantification

The sample NMR spectra were subjected to preprocessing, which involved auto-phase, auto-baseline corrections, and apodization (exponential: 0.2 Hz; stanning: 4). Chemical shifts (δ) are denoted in parts per million (ppm) and referenced to the TSP peak (δ = 0.00) using MestreNova software (version 12.0.2, Mestrelab Research, Santiago de Compostela, Spain). For metabolite identification, we utilized Chenomax NMR Suite evaluation Profiler version 9.02 (Chenomax NMR Suite 9.02, Chenomx Inc., Canada) and the coupling constant (J) values from the study by Govindaraju et al. to identify 14 distinct metabolite signals in the NMR spectra [42]. To validate metabolite identities, we cross-referenced chemical shifts and multiplicities with the Human Metabolome Database and data previously reported on rodent models [40, 43–46]. Only metabolites whose peaks were observed clearly in the spectra without overlapping with other peaks were included in the analysis. Metabolite concentrations were determined by using the integral values of the peaks, which represent the areas under the metabolite peaks. Manual integration for each peak was performed using MestreNova software. Peak and sum integration were chosen as a calculation method for the integration of metabolites and TSP peak respectively in all the sample spectra. TSP integration value was set to 1. The absolute concentration of metabolites was calculated using the TSP concentration as a reference, following the metabolite concentration formula reported by Zheng et al. (Equation 1) [45]. Finally, the concentration of identified metabolites was expressed as mmol/L.

1 $$\:\text{C}=\frac{{\text{P}}_{\text{M}}\times\:{\text{N}}_{\text{M}}\times\:{\text{C}}_{\text{T}\text{S}\text{P}}\times\:\text{V}}{{\text{P}}_{\text{T}\text{S}\text{P}}\times\:{\text{N}}_{\text{T}\text{S}\text{P}}\times\:\text{W}}$$

Where C is the concentration of metabolite; P_M_ is the peak area of metabolite; N_M_ is the proton number of metabolite; C_TSP_ is the concentration of TSP (2.62mM); V is the final sample volume (35 ml); P_TSP_ is the peak area of TSP (1); N_TSP_ is the proton number of TSP (9); W is the tissue weight.

## Data Processing and statistical analysis

### Univariate and Multivariate analysis

Excel files containing data were uploaded to the free online tool MetaboAnalyst 6.0 software [47, 48]. An unsupervised principal component analysis (PCA) and t-test were performed on weight-normalized, log-transformed, and Pareto-scaled data, a centered mean, and division by the square root of the standard deviation of each variable. PCA gave an idea of similarities (clustering between data sets) and differences (far apart data sets) between samples. A supervised partial least squares discriminant analysis (PLS-DA) was also performed, which helped in identifying metabolites contributing to the discrimination between samples. The cross-validation model helped in assessing the performance of the PLS-DA model where the Q^2^ value estimates the predictive ability of the model. Variable importance in projection (VIP) is one of the important measures in the PLS-DA model and it was used to know about the significance of variables in PLS-DA. Only metabolites with VIP > 0.5 were considered for further analysis.

### Statistical analysis

Metabolites collectively qualifying the criteria of VIP > 0.5 and fold change (FC) > 1 were statistically analyzed between WT and AS samples. Comparison of means using student’s t-tests with correction for multiple comparisons using false discovery rate (FDR) was applied, and the adjusted p-value < 0.05 was considered significant. Fold changes of differences were reported and effect sizes of significantly altered metabolites were calculated using GPower 3.1.

### Pathway topology

To identify the dysregulated pathways in AS, the metabolite concentrations of AS and WT samples were uploaded to the enrichment and pathway analysis section of Metaboanalyst 6.0 software [47, 48]. For metabolite set enrichment analysis (MSEA), the “current 2019” KEGG pathway library was used. The quantitative enrichment analysis (QEA) was performed for enrichment pathway analysis. This QEA algorithm uses a generalized linear model to calculate the *p*-values, Holm-adjusted *p*-values, and estimates of false discovery rate (FDR) for each metabolite set.

## Results

In our previous studies, we showed that NPCs from the brains of AS model mice exhibit enhanced oxidative stress and mitochondrial dysfunction. Particularly, mitochondrial membrane potential and mitochondrial ROS expression levels were increased in AS NPCs, compared to WT. These findings were correlated with enhanced levels of apoptosis in AS NPCs, and the reduction of mitochondrial oxidative stress by administering antioxidant reduced the levels of apoptosis [11]. The objective of the herein study was to uncover the altered mitochondrial metabolic profile associated with AS at the embryonic developmental stage and to better understand the downstream effects of these metabolic aberrations in the brain tissue of AS model mice. For that, we utilized ^1^H-NMR spectroscopy and conducted metabolomic analysis to delineate the changes in metabolite levels and their associated metabolic pathways.

### NMR-Based metabolic profiles of WT and AS embryonic brains show a clear separation between the two genotypes

**Fig. 1:**
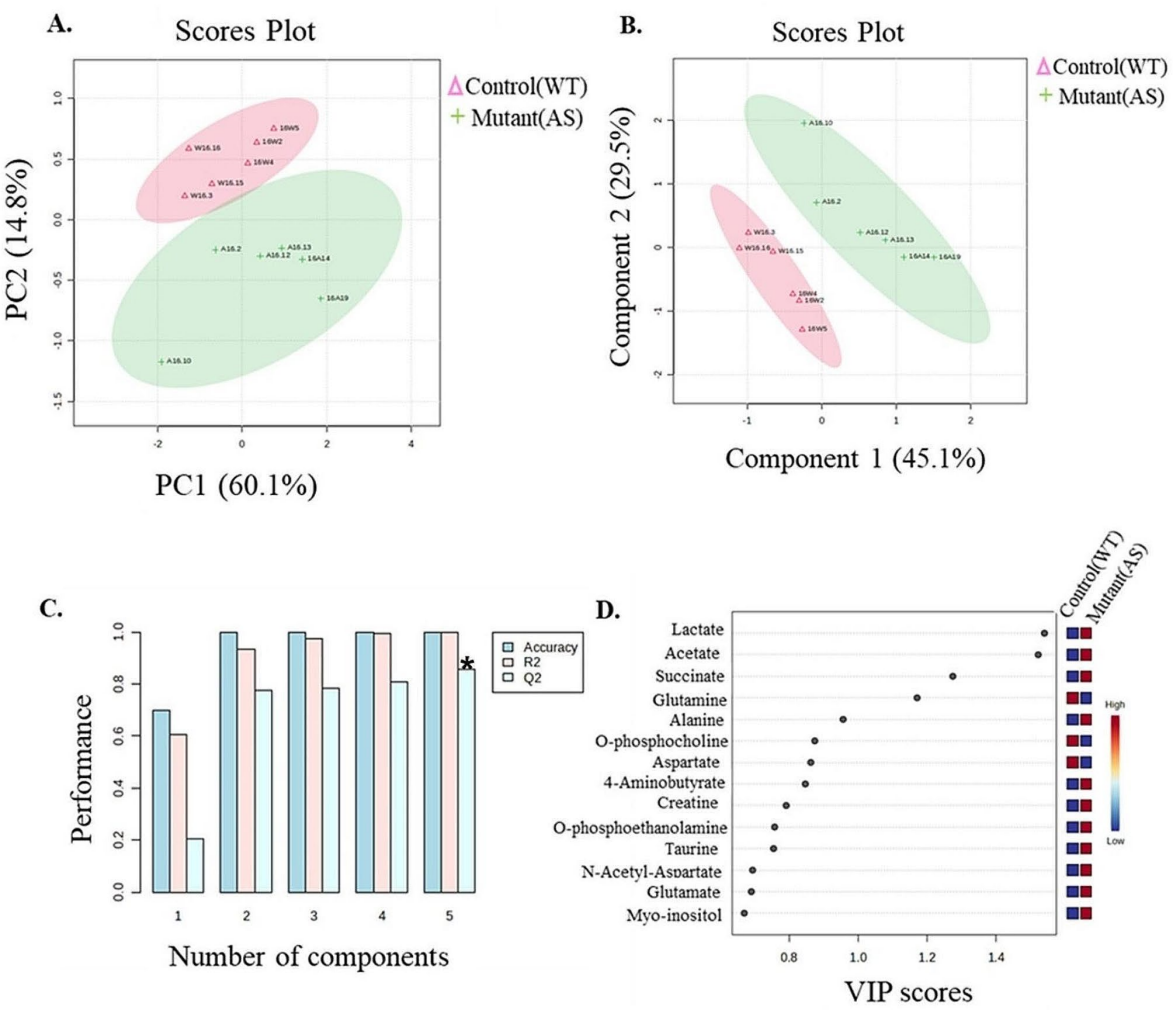
Multivariate statistical analysis distinguishing male control (WT) (pink color) and mutant (AS) (green color) mice. (**A**) PCA plot showing AS and WT samples clustered together in different regions. (**B**) PLS-DA plot showing metabolites responsible for separation between AS and WT samples. (**C**) Validation of PLS-DA model with R^2^ = 99.7%, Q^2^ = 85.0%. The asterisk denotes the particular component chosen by metaboanalyst software for the calculation of the VIP scores of the PLS-DA model. (**D**) VIP (Variable importance in projection) plot showing most important metabolites with VIP > 0.5 to be responsible for separation between AS and WT samples in PLS-DA plot. *N* = 6 per genotype

Initially, we aimed to determine the expression level of the various identifiable metabolites and examine whether their expression is altered in the brains of male AS embryos. For that, we performed ^1^H-NMR spectroscopy of the AS and WT littermate’s embryonic brains and received the ^1^H-NMR spectra of these samples (Supplementary Fig. 1A, B, and C). From these spectra, we were able to identify with high certainty 14 metabolites that were present in all samples (Supplementary Table 1), and analyzed their expression level. Metabolites whose peaks were visible and not overlapping were included in the analysis. Partially or completely overlapping peaks of metabolites were not included in the study. Our initial observation was that for most metabolites, the intensities of NMR spectra recorded from the AS samples were generally higher than those from the WT samples (supplementary Fig. 1C). Next, we wanted to examine whether the metabolite expression levels can separate between the WT and AS samples. For that, we utilized PCA, which showed a clear separation between the two groups (Fig. 1A). PCA helps in identifying similarities and differences between samples based on their overall metabolic profile. In the PCA analysis of 14 metabolites, we observed two distinct clusters of WT and AS samples, suggesting that distinct metabolic profiles were involved in the formation of these two clusters. Next, to complement the PCA, we performed PLS-DA which is used to identify which metabolites are important for distinguishing between the two genotypes. Similar to the PCA results, PLS-DA analysis (Fig. 1B) also showed a clear separation between the two genotypes, and the performance parameters of the PLS-DA clearly showed the accuracy of 1, high validity with an R^2^ = 99.7%, and a high predictive ability of the model, which was shown by calculating the cross-validation score with the estimate of Q^2^ = 85.0% (Fig. 1C, Supplementary Table 2). In addition, we performed an analysis of the VIP statistics and we chose the margin of VIP > 0.5 to identify the metabolites that are the major contributors to the separations between the two groups (Fig. 1D). All fourteen high-fidelity identified metabolites had a VIP score larger than 0.5. Of these 14 metabolites, 3 were downregulated and 11 were upregulated (Fig. 1D, Supplementary Table 3).

### AS embryonic brain shows significant elevation of three metabolites

**Fig. 2:**
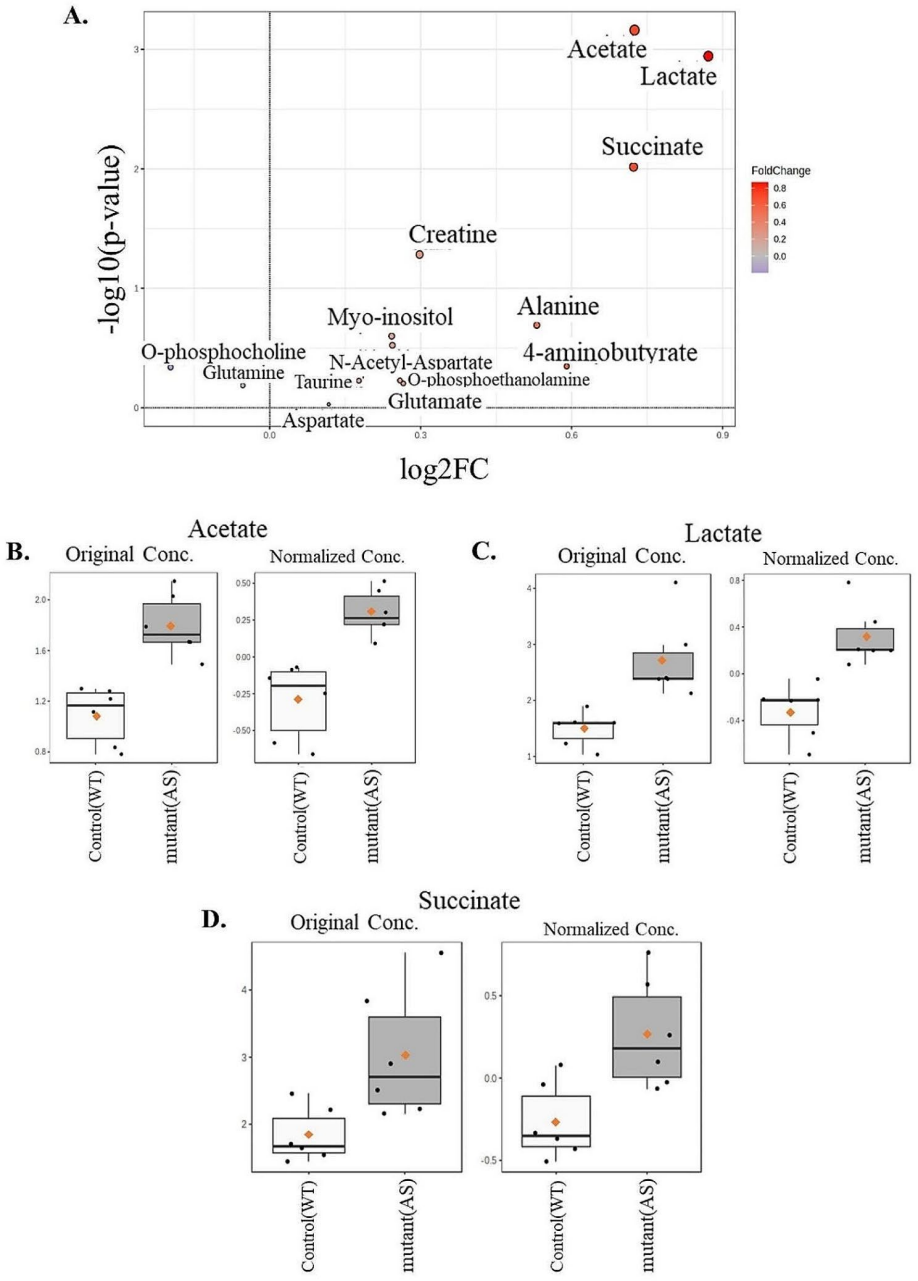
Univariate statistical analysis to compare the expression of individual metabolites in between the two genotypes. Metabolites that are upregulated in AS are shown in orange color and those downregulated are shown in blue color. (**A**) Volcano plot showing up- and down-regulated metabolites. (**B**), (**C**), & (**D**) Box plot showing the expression levels of three significantly upregulated metabolites namely, acetate, lactate, and succinate. The fold changes among the study groups were calculated using MetaboAnalyst 6.0 software. The orange dots depict the means, and the transverse black lines in the boxes depict the medians. *N* = 6 per genotype. Raw p-values following Student’s t-test: acetate (*p* = 6.886E-4), lactate (*p* = 0.0011), succinate (*p* = 0.0096). Adjusted p-values following false discovery rate correction for multiple comparisons: acetate (FDR = 0.0079561), lactate (FDR = 0.0079561), succinate (FDR = 0.044904)

Since our multivariate analysis utilizing PCA and PLS-DA model showed the important role of metabolites in separating the two genotypes, next, we wanted to identify the alteration in the level of specific metabolites in the AS samples. For this, we utilized the univariate analysis on the identified 14 metabolites, which included the volcano plot and t-test analysis (Fig. 2A; Table 1). Among the 11 upregulated metabolites, 4 metabolites were found to have *p* ≤ 0.05 in the AS samples compared to the WT samples. These four metabolites included: acetate (*p* = 6.886E-4), lactate (*p* = 0.0011), succinate (*p* = 0.0096), and creatine (*p* = 0.0521). Out of the four metabolites, three metabolites remained significant after false discovery rate (FDR) correction namely, acetate (FDR = 0.0079), lactate (FDR = 0.0079), and succinate (FDR = 0.0449). Among the three significantly upregulated metabolites, lactate showed the highest level of fold change (FC = 1.82), which was followed by acetate (FC = 1.65) and succinate (FC = 1.65) (Fig. 2B, C, D; Table 1). Additionally, the effect size of the differences in the metabolite level between the two genotypes was large (Cohen’s d ≥ 0.8). Specifically, the effect size (Cohen’s d) for the three significantly elevated metabolites were: acetate (Cohen’s d = 3.00), lactate (Cohen’s d = 2.20), succinate (Cohen’s d = 1.61) (Supplementary Table 4 A, B, and C). Furthermore, the cluster analysis utilizing the heat map also showed uniformity in the individual expression pattern of these three metabolites where almost all of the AS samples had higher expression levels in comparison to the WT samples (Supplementary Fig. 2). These findings indicate the involvement of altered metabolism in the embryonic pathogenesis of the AS.

**Table 1:** Fold change and p values of 14 metabolites in a t-test analysis

Name	FC	log2(FC)	raw.*p*-value	-log10(*p*)	FDR
Acetate	1.6529	0.72498	6.886E-4	3.162	0.0079561
Lactate	1.8297	0.87164	0.0011366	2.9444	0.0079561
Succinate	1.6505	0.72293	0.0096222	2.0167	0.044904
Creatine	1.2293	0.29785	0.05211	1.2831	0.18238
Alanine	1.4447	0.53076	0.20345	0.69154	0.56966
Myo-inositol	1.1831	0.24253	0.25132	0.59978	0.5864
N-Acetyl-Aspartate	1.1841	0.24384	0.29986	0.52308	0.59972
4-Aminobutyrate	1.5054	0.59013	0.44937	0.3474	0.70053
O-phosphocholine	0.87251	-0.19676	0.45788	0.33924	0.70053
O-phosphoethanolamine	1.1966	0.2589	0.59158	0.22798	0.70053
Taurine	1.1308	0.17734	0.5942	0.22607	0.70053
Glutamate	1.202	0.26549	0.6249	0.20419	0.70053
Glutamine	0.9637	-0.053337	0.65049	0.18676	0.70053
Aspartate	1.0848	0.11738	0.93638	0.02855	0.93638

FC, fold change; FDR, false discovery rate. *n* = 6 samples per genotype

**Fig. 3:**
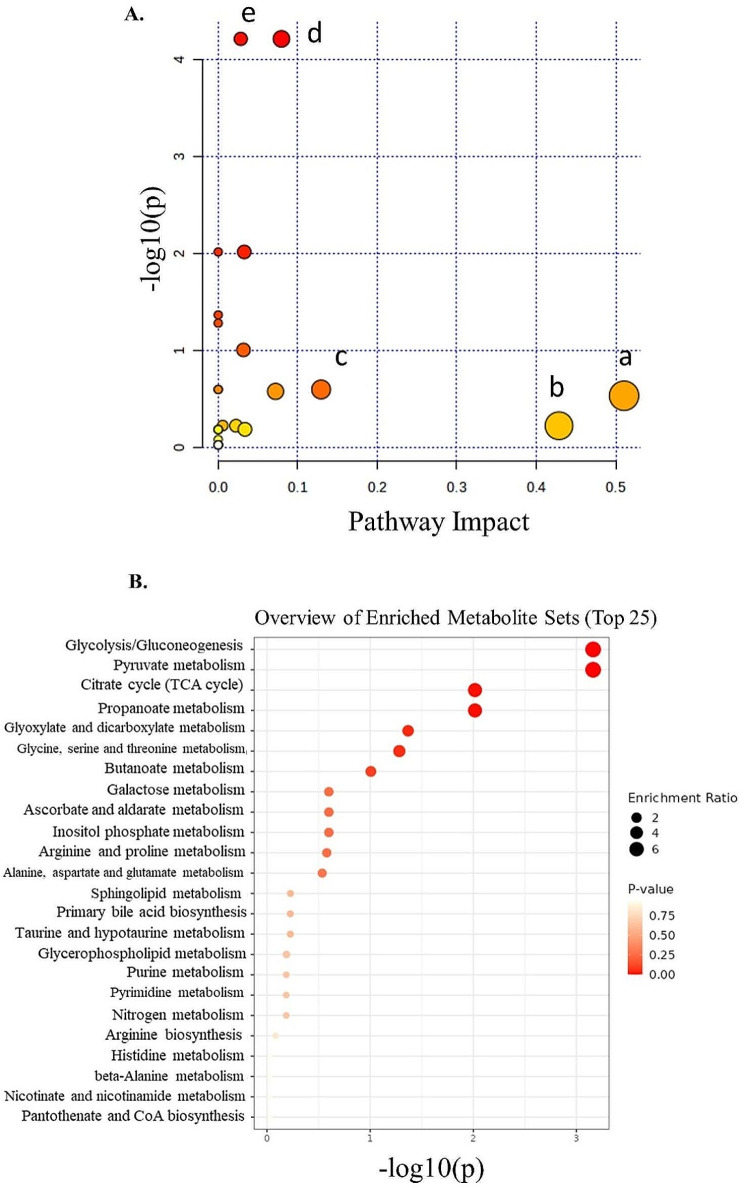
Pathway and enrichment analysis of metabolites from E16.5 embryonic brains of male AS and WT mice. (**A**) The horizontal coordinate indicates the pathway impact value, the vertical coordinate indicates the − log10(*p*) of the pathway, a dot in the figure represents a metabolic pathway, the size of the dot is proportional to its impact value, and the color of the dot represents the size of the pathway *P* value, where the color change from yellow to red represents the change in the *P* value from large to small. (a) Alanine, Aspartate and glutamate metabolism; (b) Taurine and hypotaurine metabolism; (c) Inositol phosphate metabolism; (d) Pyruvate metabolism; (e) Glycolysis / Gluconeogenesis; (f) Citrate cycle (TCA cycle); (g) Butanoate metabolism; (h) Arginine and Proline metabolism. (**B**) Dot plot of the enrichment analysis. Enrichment ratio is computed by Hits / Expected, where hits = observed hits; expected = expected hits. *N* = 6 per genotype. Raw p-values: pyruvate metabolism (*p* = 6.1455E-5), glycolysis/gluconeogenesis (*p* = 6.1455E-5), TCA cycle (*p* = 0.0096). Adjusted p-values following false discovery rate correction for multiple comparisons: pyruvate metabolism (FDR = 7.3745E-4), glycolysis/gluconeogenesis (FDR = 7.3745E-4), TCA cycle (FDR = 0.057733).

### Pyruvate metabolism and Glycolysis are affected in AS

**Fig. 4:**
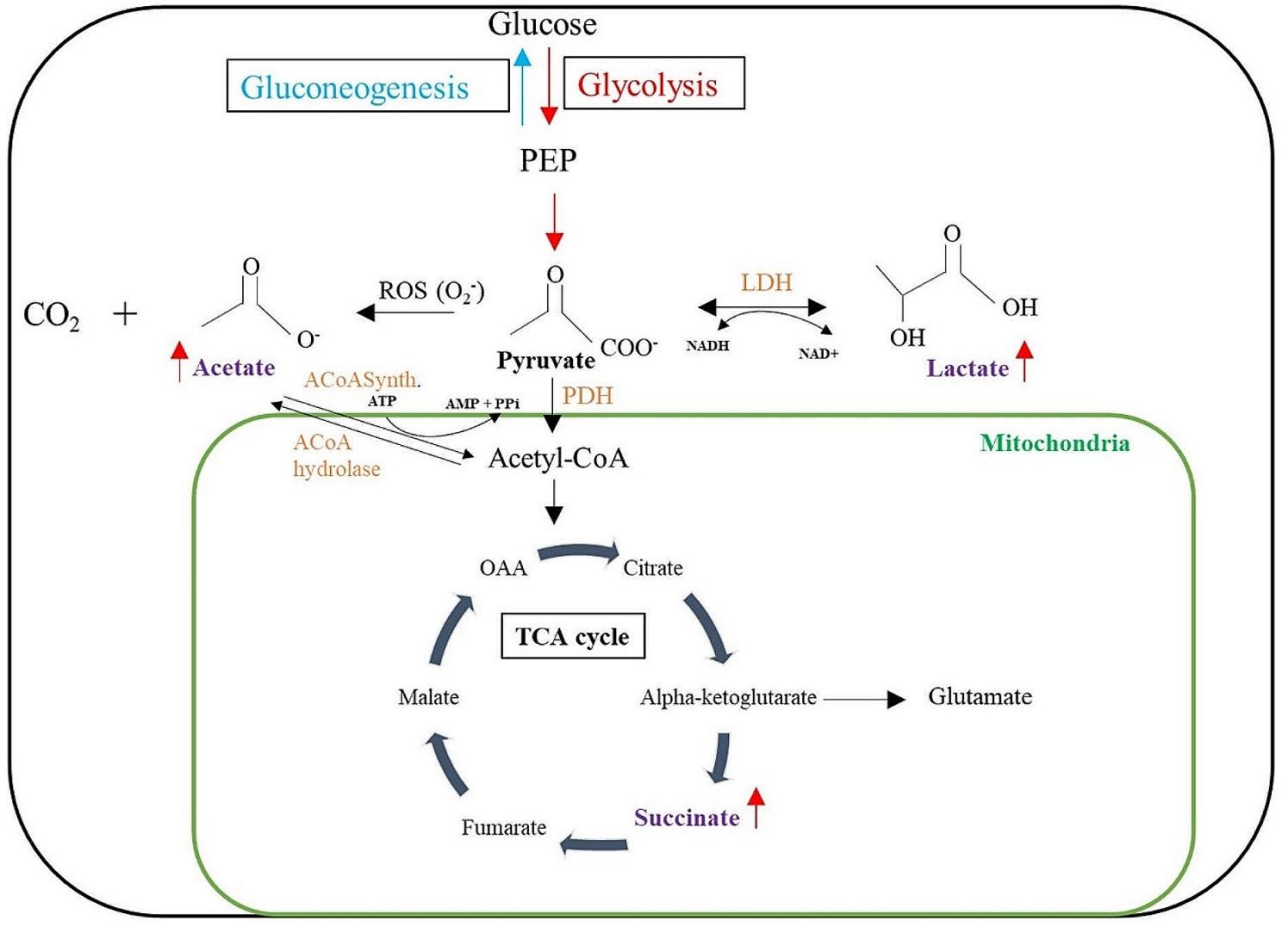
Overview of metabolites and associated metabolic pathway alteration in Angelman syndrome. Glycolysis involves the formation of pyruvate from glucose and pyruvate is converted to acetyl-CoA to enter into the TCA cycle, where succinate is one of intermediate metabolite. Pyruvate can convert into lactate or acetate in the presence of lactate dehydrogenase (LDH) or reactive oxygen species (ROS) respectively. *Abbreviations*: phosphoenol pyruvate (PEP); oxaloacetate (OAA); acetyl-coenzyme A (acetyl-CoA)

Metabolites in embryonic brain cells play a crucial role in the biological pathways during brain development and their optimum levels are required to be maintained for optimal neuronal functioning, myelin lipid synthesis, energy metabolism, and cellular signaling [49, 50]. Therefore, we wanted to investigate the molecular pathways associated with the metabolites that were found to be elevated in the present study. For this, based on the 14 identified metabolites, we performed two independent analyses, pathway analysis on the Mus musculus database and enrichment analysis on the human database (Tables 2, 3). Despite slight differences between these two analyses (Tables 2, 3), their combination yields ‘glycolysis’, ‘pyruvate metabolism’, and ‘tricarboxylic acid (TCA) cycle’ as the top three dysregulated pathways in the AS compared to the WT littermates (Fig. 3). Noteworthy, ‘glycolysis’ and ‘pyruvate metabolism’ were highly significant following FDR correction for multiple comparisons (FDR < 0.01 with mus musculus and FDR < 0.01 with human metabolic database), whereas ‘TCA cycle’ was nearing significance following FDR correction for multiple comparisons (FDR = 0.0577 for both databases) (Tables 2 and 3). From the differentially expressed metabolites both lactate and acetate are associated with the ‘glycolytic pathway’ and ‘pyruvate metabolism’, while succinate is involved as an intermediate metabolite in the ‘TCA cycle’ (supplementary Table 5). Also, these metabolites were found to be linked with mitochondrial metabolism (Fig. 4).

**Table 2:** Pathway analysis of the identified metabolites using Mus musculus dataset

Pathway Name	Match Status	*p*	-log(*p*)	Holm *p*	FDR	Impact
Pyruvate metabolism	2/23	6.1455E-5	4.2114	0.0014749	7.3745E-4	0.07951
Glycolysis / Gluconeogenesis	2/26	6.1455E-5	4.2114	0.0014749	7.3745E-4	0.02831
Citrate cycle (TCA cycle)	1/20	0.0096222	2.0167	0.21169	0.057733	0.03273
Butanoate metabolism	2/15	0.098379	1.0071	1.0	0.3373	0.03175
Inositol phosphate metabolism	1/30	0.25132	0.59978	1.0	0.57397	0.12939
Arginine and proline metabolism	2/36	0.26307	0.57993	1.0	0.57397	0.07209
Alanine, aspartate and glutamate metabolism	5/28	0.29155	0.53529	1.0	0.58309	0.51042
Sphingolipid metabolism	1/32	0.59158	0.22798	1.0	0.82167	0.00563
Taurine and hypotaurine metabolism	1/8	0.5942	0.22607	1.0	0.82167	0.42857
Primary bile acid biosynthesis	1/46	0.5942	0.22607	1.0	0.82167	0.02239
Glycerophospholipid metabolism	2/36	0.64707	0.18905	1.0	0.82167	0.0336

Pathway ‘impact value’ represents the combination of the centrality (biological relevance) of the metabolites within the pathway and pathway enrichment results (statistical significance). ‘Match status’ refers to the association between the metabolites in the experimental data and the metabolites annotated in a particular pathway in the Mus musculus metabolic dataset. FDR, false discovery rate

**Table 3:** Metabolite Set Enrichment Analysis (MSEA) of the identified metabolites using human dataset

Metabolite Set	Total	Hits	Statistic	Expected	*P* value	Holm *P*	FDR
Glycolysis / Gluconeogenesis	26	1	70.023	9.0909	6.886E-4	0.016526	0.0082632
Pyruvate metabolism	23	1	70.023	9.0909	6.886E-4	0.016526	0.0082632
Citrate cycle (TCA cycle)	20	1	50.467	9.0909	0.0096222	0.21169	0.057733
Propanoate metabolism	21	1	50.467	9.0909	0.0096222	0.21169	0.057733
Glyoxylate and dicarboxylate metabolism	31	2	26.143	9.0909	0.04286	0.8572	0.20573
Glycine, serine and threonine metabolism	33	1	32.689	9.0909	0.05211	0.99009	0.20844
Butanoate metabolism	15	2	21.555	9.0909	0.098379	1.0	0.3373
Galactose metabolism	27	1	12.912	9.0909	0.25132	1.0	0.57397
Ascorbate and aldarate metabolism	9	1	12.912	9.0909	0.25132	1.0	0.57397
Inositol phosphate metabolism	30	1	12.912	9.0909	0.25132	1.0	0.57397
Arginine and proline metabolism	36	2	12.014	9.0909	0.26307	1.0	0.57397
Alanine, aspartate and glutamate metabolism	28	5	10.91	9.0909	0.29155	1.0	0.58309
Sphingolipid metabolism	32	1	2.9804	9.0909	0.59158	1.0	0.82167
Primary bile acid biosynthesis	46	1	2.9391	9.0909	0.5942	1.0	0.82167
Taurine and hypotaurine metabolism	8	1	2.9391	9.0909	0.5942	1.0	0.82167

‘Total’ refers to the total number of metabolites present in a particular pathway of human metabolome dataset. ‘Hit’ refers to the number of metabolites of a particular pathway present in the experimental data. FDR, false discovery rate

## Discussion

In this study, we investigated the changes in the level of metabolites in the AS brain at an embryonic stage E16.5. Embryonic day (E16) marks the end of neurogenesis in the mice brain development [51]. By employing NMR spectroscopy-derived global metabolome profiling, we aimed to identify alteration in the level of specific metabolites and associated biological pathways in the AS brain during embryonic development and to gain a better understanding of the dysregulated molecular pathways associated with the loss of function of the UBE3A protein.

Our multivariate analysis, utilizing both the PCA and PLS-DA models, showed a separation between WT and AS samples (Fig. 1A and B). Additionally, the PLS-DA model identified 14 crucial metabolites (VIP < 0.5) responsible for distinguishing between mutant (AS) and control (WT) samples. Notably, the distinct clusters were observed for both sample types in the plot, emphasizing the importance of these identified metabolites in elucidating the metabolomic differences between the WT and AS conditions. Amongst these 14 metabolites, three (acetate, lactate, and succinate) were found to be significantly upregulated in the AS samples.

Neural energy metabolism of the embryonic brain is different from the adult. During the perinatal period of embryonic brain development, lactate is utilized as a main source of energy. The source of lactate can be either blood-derived from the placenta or the conversion of pyruvate [52, 53]. However, in the adult brain, the lactate is known to originate from astrocytes via the astrocyte-lactate shuttle [53]. Lactate serves as an alternative energy source for the brain, especially when glucose availability is limited [54, 55]. Lactate is produced through anaerobic glycolysis, by the conversion of pyruvate to lactate in the presence of lactate dehydrogenase (LDH) [56]. During development, lactate is known to play a role in cell proliferation and differentiation [52]. Elevated lactate levels were observed in neurological disorders like autism spectrum disorder (ASD) and schizophrenia, potentially linked to a shift from the TCA cycle to glycolysis [32, 33, 57].

In this study, abnormal lactate levels, notably around 83% (highest fold change among all other metabolites), were found in the AS samples, suggesting disrupted brain energy metabolism. During early embryonic development, increased mitochondrial oxidative phosphorylation is essential for cell differentiation, requiring a transition from anaerobic to aerobic metabolism, leading to lower lactate production [58]. Abnormally high lactate levels in the AS samples indicate dysfunctional energy metabolism, possibly due to reduced oxygen supply (hypoxia) or reduced ability to utilize mitochondrial TCA cycle resulting in enhanced aerobic glycolysis or altered cellular differentiation [59].

Elevated lactate levels can be further accompanied by the observation of abnormally high levels of TCA cycle intermediate, succinate in the AS samples. Succinate is a dicarboxylic acid that plays a crucial role in energy metabolism and the mitochondrial TCA cycle. The TCA cycle, also known as the Citric acid cycle or Krebs cycle, is a central pathway in cellular respiration where succinate is formed from Succinyl-CoA. Succinate dehydrogenase (SDH) is an enzyme that converts succinate to fumarate in the TCA cycle. SDH activity has been suggested to be associated with ROS production [60]. Succinate is involved in the production of ATP and energy metabolism [61]. Altered levels of succinate have also been reported in other neurodevelopmental and neurological disorders, such as attention deficit hyperactive disorder (ADHD), neonatal hypoxia, Leigh syndrome, and SDH Deficiency [34, 62–65]. Dysregulated TCA cycle metabolites could play a role in the development of abnormal energy metabolism in the AS pathophysiology. Similar to lactate, increased succinate levels have also been associated with hypoxia [60].

In the ^1^H-NMR spectra, the creatine peak represents both creatine and creatine phosphate. Creatine converts to creatine phosphate which acts as a reservoir for the generation of ATP in the cytosol of muscle and brain tissues [42, 66]. Changes in creatine levels have been observed in various neurological and genetic disorders such as Alzheimer’s disease, depression, and myotonic dystrophy. These alterations are attributed to the increased glial content or decreased uptake of creatine by neurons [67–69]. A tendency towards higher creatine levels in the AS was observed, although this did not reach statistical significance (*p* = 0.052). This trend for alteration in creatine levels indicates and further emphasizes the important role of neurochemical changes in the dysregulation of energy metabolism in Angelman syndrome. Since our 1D NMR technique was limited in its ability to distinguish between the two peaks of creatine, future studies using the 2D NMR technique or 1D NMR devices with higher magnetic field intensities and higher resolution could distinguish between the two peaks and resolve this issue. Such future studies that will analyze the different peaks of creatine and creatine phosphate might provide more details about the involvement of energy metabolism in AS pathogenesis.

Acetate, in the form of acetyl-coenzyme A (Ac-CoA), is present in the mitochondria serving as a carbon donor in the TCA cycle, production of citrate, and generation of ATP [70, 71]. Increased acetate has also been found in other neurodevelopmental disorders and cerebral ischemia [34, 72]. A recent study by Liu et al. has found that acetate production can also occur through a pathway independent of acetyl-CoA [73]. This pathway involves the direct conversion of pyruvate to acetate. Pyruvate is a keto acid with electrophilic moiety. One of the pathways involves ROS generation where in the presence of oxygen and hydrogen peroxide, superoxide radicals are formed which carry out nucleophilic attack on pyruvate that leads to the formation of acetate. In brief, endogenous hydrogen peroxide was shown to be involved in the formation of acetate from pyruvate [73]. This may indicate a plausible direct metabolic consequence of increased ROS levels observed during embryonic development in the AS [11].

Among the pathways associated with the significantly altered metabolites, pyruvate metabolism, and glycolysis/gluconeogenesis were found to involve both the lactate and acetate metabolites. Pyruvate is an intermediate compound in the metabolism of fats, proteins, and carbohydrates. In pyruvate metabolism, pyruvate can be formed from glucose via glycolysis and then in conditions of insufficient oxygen or cells with few mitochondria, pyruvate is reduced to lactate to re-oxidize NADH back into NAD+. Pyruvate can be converted into carbohydrates via gluconeogenesis, to fatty acids or energy through acetyl-CoA via the TCA cycle [74]. Aberrant pyruvate metabolism is associated with cancer and neurodegenerative disorders [75].

In addition, we have also found the TCA cycle to be one of the affected pathways in AS. Altered TCA cycle activity has also been observed in Alzheimer’s disease and autism [31, 76].

Overall, the elevated levels of lactate, succinate, and acetate and the involvement of glycolysis and TCA cycle in their regulation indicate a possible metabolic mechanism involved in the dysregulation of mitochondrial functioning and production of ROS observed previously in the AS that occurs early during embryonic development [11, 18, 20]. Other studies of ASD-like syndromes pointed to mitochondrial dysregulation via perturbation of myo-inositol and glutamate levels in brains of mice with ASD induced using valproic acid compared to additional ASD studies, showing variation in metabolites [77].

Although it is evident from previous studies by us and others, that UBE3A plays a role in mitochondrial functioning, its actual role is yet unknown [11, 19, 22]. UBE3A function is twofold, serving as an E3-ligase and a steroid hormone receptor co-agonist, especially for androgens and estrogens [78, 79]. Unfortunately, the targets of UBE3A are mostly unknown. Hence, it is possible that UBE3A regulates the expression of mitochondrial-related proteins, thus indirectly regulating mitochondrial function. Furthermore, it is also possible that UBE3A regulates the expression of enzymes or regulatory proteins that are related to metabolism outside the mitochondria, regulating the levels of various metabolites and indirectly modulating mitochondrial functioning by affecting their accessibility to the mitochondria [80, 81].

Our results indicate the possible involvement of *Ube3a* in regulating mitochondrial metabolic functioning and suggest the involvement of glycolysis and energy metabolism alterations during the embryonic development of AS. Such changes probably are related to the enhanced apoptosis levels we previously observed [11]. It is possible that this further induces either a delay or an aberration in the metabolic switch between anaerobic glycolysis to oxidative phosphorylation that is required for the development of mature neurons, and possibly alters the developmental trajectory of embryonic brain cells [28–30]. This study, utilizing NMR techniques, represents the first to identify metabolic alterations at the late embryonic stage of a neurodevelopmental disorder.

## Limitations

Acknowledging the study limitations, ^1^H NMR, while unique in its ability to quantitate absolute metabolite concentrations, faces challenges with overlapping peaks and unidentified metabolites. As a result, only 14 metabolites could be successfully identified and investigated in our study. To gain a deeper understanding across a wider spectrum of metabolites, future research will incorporate complementary spectroscopic techniques and will employ higher NMR fields for higher sensitivity and resolution. Furthermore, our study investigated the metabolic alterations in tissue samples that included the whole embryonic brain. It is possible that spatial high-resolution studies that focus on distinct brain regions could reveal additional metabolic differences between brain regions in the developing AS brain. Moreover, the combination of such sophisticated techniques with high-resolution spatial metabolomic analyses using mass spectrometry with the addition of single-cell transcriptome analyses could be more informative concerning the metabolic changes in different cell types. Another limitation of the study is its sample size. We used a sample of 6 male mice per genotype which was sufficient considering the power analysis for the reported metabolites (Supplementary Table 4) and we reported their corresponding Cohen’s-d effect sizes. Yet, it is possible that increasing the sample size would result in additional significant metabolites, such as creatine.

## Conclusions

Despite the modest sample size, our study aligns with previous research. In conclusion, our univariate analysis highlights three significantly dysregulated metabolites in AS mouse brain samples during embryonic development (E16.5), marking the first report of metabolic profile alterations in AS at this stage. We have found altered levels of mitochondrial-related metabolites and associated metabolic pathways during the developmental stage of AS. Alteration in the TCA and glycolytic cycle metabolites such as succinate, lactate, and acetate point towards the abnormal brain energy metabolism in the AS. All the observed altered metabolites were found to be linked with mitochondrial ROS and the observed increased levels of acetate could be the consequence of increased ROS production in the AS during embryonic development. Furthermore, these altered metabolic profiles may sway brain cells from their original fate by affecting their proliferation, differentiation, or apoptosis, thus disrupting brain development [28, 82–84]. Additional studies investigating developmental delays as metabolic disorders in other study designs could build on the present findings confirm results, and point to the development of potential treatments to mitigate challenging symptoms.

### Electronic supplementary material

Below is the link to the electronic supplementary material.

Supplementary Material 1

Supplementary Material 2

Supplementary Material 3

Supplementary Material 4

Supplementary Material 5

## Data Availability

The datasets used for analyses are available from the corresponding author upon request.

## Abbreviations

Ac-CoA: acetyl-coenzyme A
ASD: autism spectrum disorder
AS: Angelman syndrome
ATP: adenosine triphosphate
D2O: deuterium oxide
E6AP: E6-associated protein
E16: embryonic day
FC: fold change
FDR: false discovery rate
GSH-EE: glutathione-reduced ethyl ester
^1^H-NMR: proton nuclear magnetic resonance
KEGG: kyoto encyclopedia of genes and genomes
LDH: lactate dehydrogenase
MEF: mouse embryonic fibroblast
MSEA: metabolite set enrichment analysis
NPCs: neural precursor cells
PCA: principal component analysis
PLS-DA: partial least squares discriminant analysis
QEA: quantitative enrichment analysis
ROS: reactive oxygen species
SDH: succinate dehydrogenase
TCA: tricarboxylic acid
TSP: trimethylsilyl-2,2,3,3, -tetradeuteroproprionate
UBE3A: ubiquitin ligase E3A
VIP: Variable importance in projection
WT: wild-type
