# Supplementary figures and images for: 1H-NMR-based metabolomics reveals metabolic alterations in early development of a mouse model of Angelman syndrome
Source: Mol Autism. 2024 Jul 24;15:31. doi: 10.1186/s13229-024-00608-2 (PMC11267930; doi:10.1186/s13229-024-00608-2)

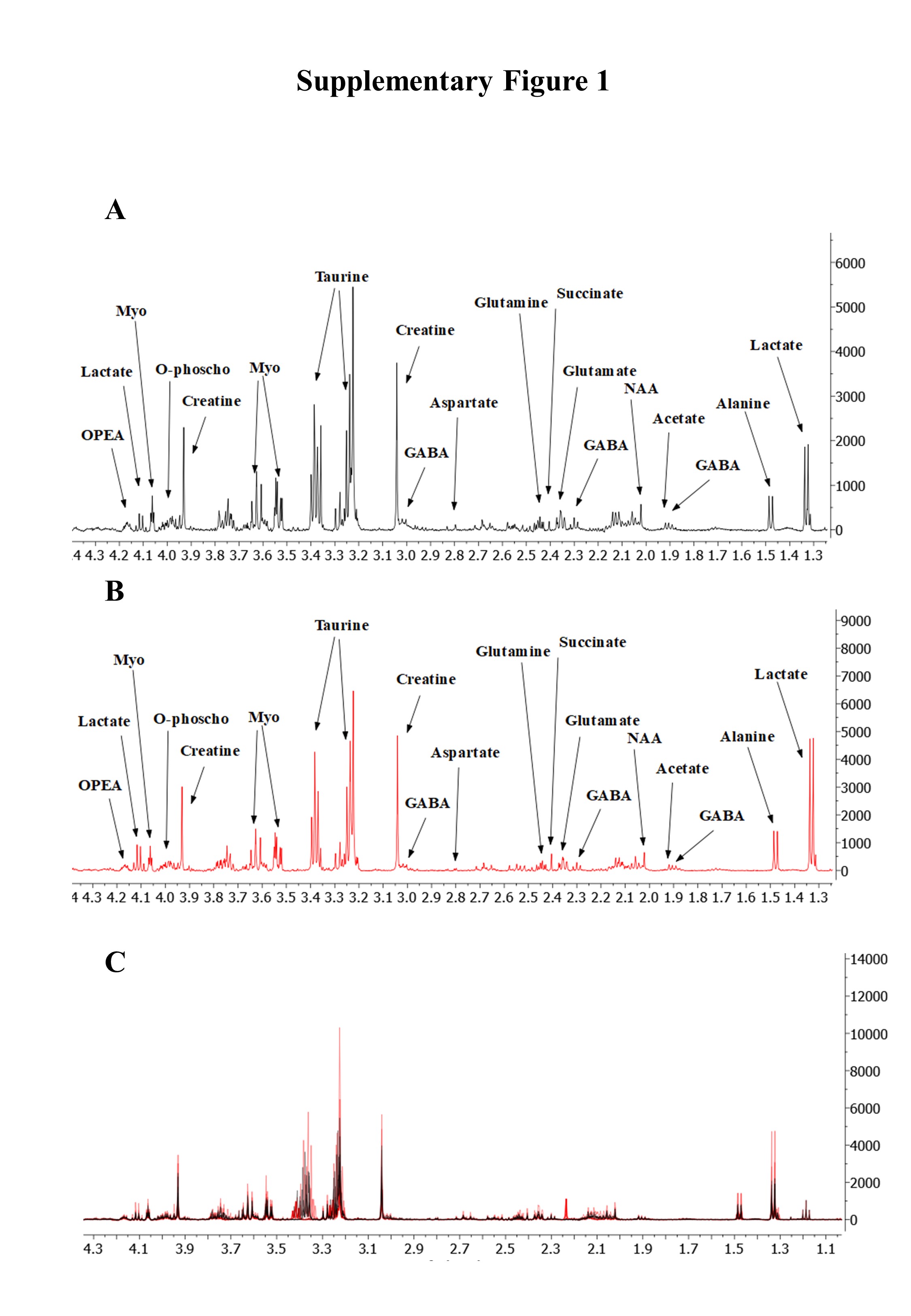

Supplement: Supplementary file 1 — Supplementary Material 1 [file 13229_2024_608_MOESM1_ESM.jpg]

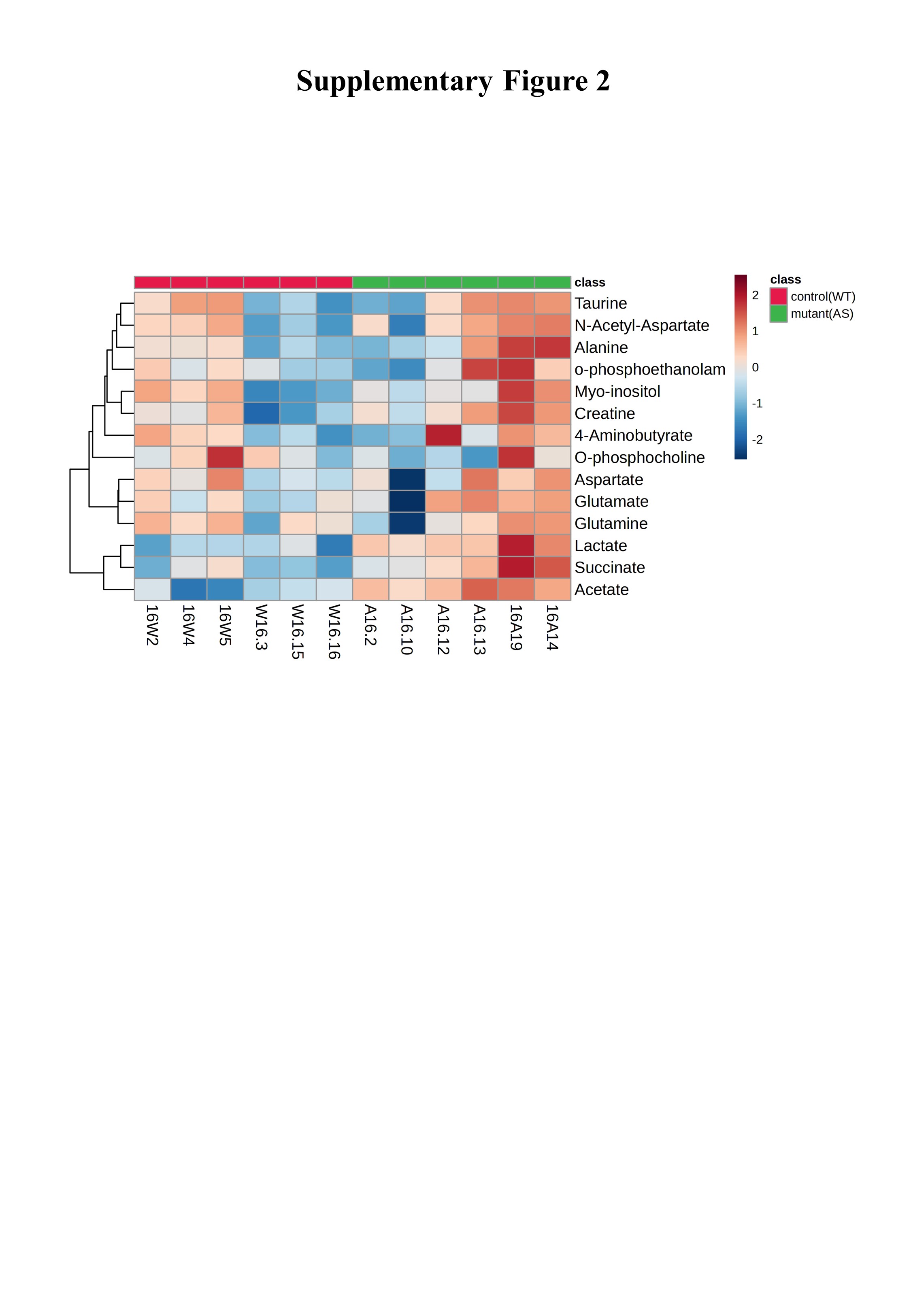

Supplement: Supplementary file 2 — Supplementary Material 2 [file 13229_2024_608_MOESM2_ESM.jpg]
