# Supplementary material for: 1H-NMR-based metabolomics reveals metabolic alterations in early development of a mouse model of Angelman syndrome
Source: Mol Autism. 2024 Jul 24;15:31. doi: 10.1186/s13229-024-00608-2 (PMC11267930; doi:10.1186/s13229-024-00608-2)
Supplement: Supplementary file 3 — Supplementary Material 3 [file 13229_2024_608_MOESM3_ESM.docx]

**Supplementary Figure Legends**

**Supplementary Figure 1. Typical 500 MHz 1H-NMR spectrum of mouse brain tissue at an embryonic stage E16.5.** (A) Wild-type (WT) mouse spectrum (black color). (B) Angelman syndrome (AS) mouse spectrum (red color). (C) Superimposed spectra of WT and AS mice. X-axis represents the chemical shift in ppm and y-axis represents the intensity.

**Supplementary Figure 2. Heatmap showing expression pattern of individual metabolites in each of the WT (red color) and AS (green color) samples.** N=6 samples per genotype.
