# Supplementary material for: 1H-NMR-based metabolomics reveals metabolic alterations in early development of a mouse model of Angelman syndrome
Source: Mol Autism. 2024 Jul 24;15:31. doi: 10.1186/s13229-024-00608-2 (PMC11267930; doi:10.1186/s13229-024-00608-2)
Supplement: Supplementary file 4 — Supplementary Material 4 [file 13229_2024_608_MOESM4_ESM.docx]

**Supplementary tables**

**Supp. Table 1**

| **S.No.** | **Metabolite** | **^1^H (ppm)** | **Position** | **Multiplicity** | **Peak appearance** |
| --- | --- | --- | --- | --- | --- |
| 1. | 4-aminobutyrate | 1.90 | β-CH_2_ | tt | POL |
|  |  | 2.3 | CH_2_ | t | OBS |
|  |  | 3.0 | CH_2_ | dt | POL |
| 2. | Acetate | 1.91 | α-CH_3_ | s | POL |
| 3. | Alanine | 1.47 | β-CH_3_ | d | OBS |
| 4. | Aspartate | 2.66 | β-CH_2_ | dd | POL |
|  |  | 2.81 | β-CH_2_ | dd | OBS |
|  |  | 3.89 | α-CH | dd | OL |
| 5. | O-phosphocholine | 3.21 | (CH_3_)_3_ | s | POL |
|  |  | 3.58 | CH_2_ | dt | OL |
|  |  | 4.16 | CH_2_ | dt | OL |
| 6. | Creatine | 3.04 | CH_3_ | s | OBS |
|  |  | 3.93 | CH_2_ | s | OBS |
| 7. | Glutamate | 2.10 | β-CH_2_ | td | POL |
|  |  | 2.35 | γ-CH | dt | OBS |
| 8. | Glutamine | 2.10 | β-CH_2_ | td | POL |
|  |  | 2.44 | γ-CH | dt | OBS |
| 9. | Lactate | 1.32 | CH_3_ | d | OBS |
|  |  | 4.10 | CH | q | OBS |
| 10. | Myo-inositol | 3.27 | CH | t | POL |
|  |  | 3.53 | (CH)×2 | dd | POL |
|  |  | 3.62 | (CH)×2 | t | POL |
|  |  | 4.06 | CH | t | OBS |
| 11. | N-acetylaspartate | 2.02 | CH_3_ | s | POL |
|  |  | 2.52 | β-CH_2_ | dd | OL |
|  |  | 2.66 | β-CH_2_ | dd | OL |
| 12. | O-Phosphoethanolamine | 3.98 | α-CH_2_ | dt | OBS |
| 13. | Succinate | 2.40 | (CH_2_)_2_ | s | OBS |
| 14. | Taurine | 3.23 | α-CH_2_ | t | POL |
|  |  | 3.36 | β-CH_2_ | t | OBS |

| Measure | 1 comps | 2 comps | 3 comps | 4 comps | 5 comps |
| --- | --- | --- | --- | --- | --- |
| Accuracy | 0.86667 | 0.93333 | 0.93333 | 1 | 1 |
| R2 | 0.60551 | 0.9348 | 0.97572 | 0.99578 | 0.99739 |
| Q2 | 0.25235 | 0.77507 | 0.79268 | 0.81234 | 0.85011 |

**Supp. Table 2**

**Supp. Table 3**

| **Name** | **Comp. 5** |
| --- | --- |
| Lactate | 1.54 |
| Acetate | 1.5219 |
| Succinate | 1.2744 |
| Glutamine | 1.1709 |
| Alanine | 0.95646 |
| O-phosphocholine | 0.87422 |
| Aspartate | 0.86262 |
| 4-Aminobutyrate | 0.84678 |
| Creatine | 0.79134 |
| o-phosphoethanolamine | 0.7578 |
| Taurine | 0.75481 |
| N-Acetyl-Aspartate | 0.69399 |
| Glutamate | 0.69027 |
| Myo-inositol | 0.66977 |

| t tests - Means: Difference between two independent means (two groups) for lactate metabolite | | | |
| --- | --- | --- | --- |
| Analysis: | Post hoc: Compute achieved power | | |
| Input: | Tail(s) | = | Two |
|  | Effect size d | = | 2.20775 |
|  | α err prob | = | 0.05 |
|  | Sample size group 1 | = | 6 |
|  | Sample size group 2 | = | 6 |
| Output: | Noncentrality parameter δ | = | 3.823935 |
|  | Critical t | = | 2.228139 |
|  | Df | = | 10 |
|  | Power (1-β err prob) | = | 0.930204 |

**Supp. Table 4**

| t tests - Means: Difference between two independent means (two groups) for acetate metabolite | | | |
| --- | --- | --- | --- |
| Analysis: | Post hoc: Compute achieved power | | |
| Input: | Tail(s) | = | Two |
|  | Effect size d | = | 3.002154 |
|  | α err prob | = | 0.05 |
|  | Sample size group 1 | = | 6 |
|  | Sample size group 2 | = | 6 |
| Output: | Noncentrality parameter δ | = | 5.199883 |
|  | Critical t | = | 2.228139 |
|  | Df | = | 10 |
|  | Power (1-β err prob) | = | 0.996481 |

**A.**

**B.**

| t tests - Means: Difference between two independent means (two groups) for succinate metabolite | | | |
| --- | --- | --- | --- |
| Analysis: | Post hoc: Compute achieved power | | |
| Input: | Tail(s) | = | Two |
|  | Effect size d | = | 1.613305 |
|  | α err prob | = | 0.05 |
|  | Sample size group 1 | = | 6 |
|  | Sample size group 2 | = | 6 |
| Output: | Noncentrality parameter δ | = | 2.794326 |
|  | Critical t | = | 2.228139 |
|  | Df | = | 10 |
|  | Power (1-β err prob) | = | 0.712166 |

**C.**

**Supp. Table 5**

| **S.No.** | **Pathway name** | **Metabolites associated with the pathway** |
| --- | --- | --- |
| 1. | Alanine, Aspartate and glutamate metabolism | Succinate, GABA, L-Glutamine, L-Glutamate, L-Alanine, L-Aspartate, N-acetylaspartate |
| 2. | Taurine and Hypotaurine metabolism | Taurine |
| 3. | D-Glutamine and D-Glutamate metabolism | L-Glutamine and L-Glutamate |
| 4. | Arginine and proline metabolism | Creatine, GABA, L-Glutamate |
| 5. | Butanoate metabolism | Succinate,GABA, L-Glutamate |
| 6. | Inositol phosphate metabolism | Myo-Inositol |
| 7. | Citrate cycle (TCA cycle) | Succinate |
| 8. | Phosphatidylinositol signaling system | Myo-Inositol |
| 9. | Primary bile acid biosynthesis | Taurine |
| 10. | Glutathione metabolism | L-Glutamate |
| 11. | Pyruvate metabolism | Lactate and Acetate |
| 12. | Arginine biosynthesis | L-Glutamate, L-Glutamine, L-Aspartate |
| 13. | Glycolysis/Gluconeogenesis | Lactate, Acetate and ethanol |
| 14. | Glycerophospholipid metabolism | Choline |
| 15. | Sphingolipid metabolism | Ethanolamine phosphate |
| 16. | Propanoate metabolism | Succinic acid |
| 17. | Glyoxylate and dicarboxylate metabolism | Acetic acid and Glutamine |
| 18. | Galactose metabolism | Myo-inositol |
| 19. | Ascorbate and aldarate metabolism | Myo-inositol |
