# Supplementary material for: 1H-NMR-based metabolomics reveals metabolic alterations in early development of a mouse model of Angelman syndrome
Source: Mol Autism. 2024 Jul 24;15:31. doi: 10.1186/s13229-024-00608-2 (PMC11267930; doi:10.1186/s13229-024-00608-2)
Supplement: Supplementary file 5 — Supplementary Material 5 [file 13229_2024_608_MOESM5_ESM.docx]

**Supplementary table legend**

**Supplementary Table 1. Characterization of 1H-NMR signals for reference metabolites.** OBS observed in male AS mice embryonic brain extracts; POL partially overlapped by other resonances; OL overlapped by other signals and unable to positively identify; s singlet; d doublet; t triple; dd doublet of doublet; m multiplet. Identified metabolites accounted for 78% of total NMR signal.

**Supplementary Table 2. PLS-DA cross-validation details.** Q2 is an estimate of the predictive ability of the model, and is calculated via cross-validation (CV). R2 provides a measure of model fit to the original data. Comps is the component number of the model.

**Supplementary Table 3. VIP score plot table showing VIP scores of 14 metabolites identified in embryonic brain tissue.** Component 5 data is shown as it was the best classifier among all components.

**Supplementary Table 4. Effect size (Cohen’s d) of three significantly upregulated metabolites calculated using GPower 3.1.** (A) Effect size d of acetate metabolite showing power (1-β) and sample size. (B) Effect size d of lactate metabolite showing power (1-β) and sample size. (C) Effect size d of succinate metabolite showing power (1-β) and sample size.

**Supplementary Table 5. The metabolic pathways that are associated with the important metabolites in pathway analysis.** Metabolites that are involved in specific pathway are listed in the table.
